# *In situ* investigation of ruthenium doped lanthanum nickel titanium double perovskite and its exsolution behaviour†

**DOI:** 10.1039/d4na00349g

**Published:** 2024-07-08

**Authors:** Jia Guo, Andrey Berenov, Stephen J. Skinner

**Affiliations:** a Department of Materials, Imperial College London Exhibition Road London SW7 2AZ UK s.skinner@imperial.ac.uk; b International Institute for Carbon Neutral Energy Research, Kyushu University Fukuoka Japan

## Abstract

Exsolution, an innovative method for fabricating perovskite-based oxides decorated with metal nanoparticles, has garnered significant interest in the fields of catalyst fabrication and electrochemical devices. Although dopant exsolution from single perovskite structures has been extensively studied, the exsolution behaviour of double perovskite structures remains insufficiently understood. In this study, we synthesized B-site double perovskite Ru-doped lanthanum nickel titanates with a 7.5 at% A-site deficiency, and systematically investigated the exsolution process that formed nickel metal nanoparticles on the material surface, across a broad reduction temperature range of 350–1000 °C. Both *Ex situ* and *in situ* characterization revealed that small, uniform Ni nanoparticles exsolved at low temperatures, whereas the exsolution of ruthenium required higher reduction temperatures beyond 1000 °C. Within the reduction temperature range of 350–500 °C, a notable finding is the reconstruction of exsolved nanoparticles, implying that Ni particles exist in a thermodynamically metastable state. Electrochemical impedance spectroscopy (EIS) showed a decreased area specific resistance (ASR) during the progress of exsolution. The increase in current density of a full solid oxide cell (SOC) in electrolysis mode and the doubling of peak power density in fuel cell mode attributed to the exsolution of Ni nanoparticles highlight the potential application of metal exsolution in electrode materials for SOCs.

## Abstract 2

Ru-doped lanthanum nickel titanate exsolution behaviour was revealed through *in situ* and *ex situ* observation. Uniform exsolved Ni nanoparticles enhanced the performance of solid oxide cells.

## Graphical abstract

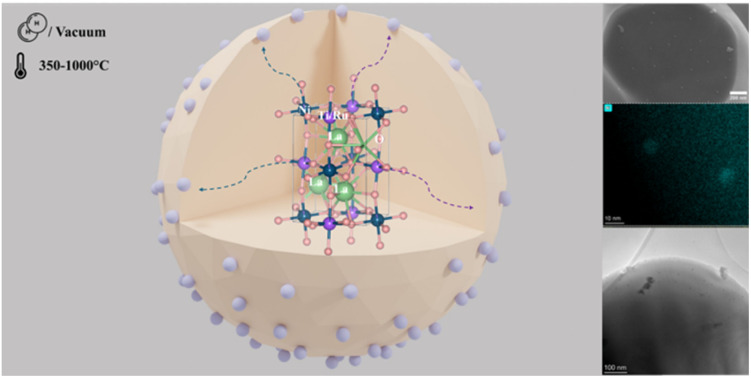

## 1. Introduction

As fossil fuel consumption continues at high levels and global climate change accelerates, there is an increasing demand for green energy alternatives needed to mitigate and reduce carbon emissions. For example, the UK government has set a target to reduce carbon emissions and achieve ‘net zero’ by 2050.^1,2^ To achieve this goal, leveraging hydrogen emerges as one of the most promising strategies. As such, solid oxide cells (SOCs), including solid oxide fuel cells (SOFC) and solid oxide electrolysis cells (SOEC), have become attractive options to transition to low carbon energy due to their high efficiency, long lifespan, low emissions, and fuel flexibility.^3,4^ Over the past decade, perovskite oxides decorated with *in situ* exsolved metal nanoparticles have been identified as promising electrodes for SOCs, with reported enhancements in overall electrochemical performance of up to 2–3 times compared to pristine perovskite electrodes.^5^ The technique employed to fabricate such nanoparticle decorated electrodes is commonly referred to as ‘exsolution’.

Exsolution includes multiple steps including reduction-induced oxygen vacancy generation within the oxide lattice, subsequent cation migration, nucleation, and nanoparticle growth.^6,7^ Studies on exsolution gained particular attention after Neagu *et al.*^8^ proposed controlling and tuning exsolved nanoparticles, promoted through the presence of A-site deficiency in a perovskite lattice. The presence of A-site deficiency facilitates the spontaneous exsolution of localized B-site ‘excess’ to restore local stoichiometry, and also suppresses the structural decomposition of the perovskite into the component oxides. A subsequent investigation into the interface between exsolved Ni nanoparticles and the lanthanum strontium titanate bulk substrate revealed a unique ‘socketed’ structure, which contributed to high thermal stability and excellent coking resistance, without sacrificing catalytic activity.^9^ These findings prompted a surge of interest in exsolved nanoparticles across diverse applications in catalyst development and electrochemical devices, including SOCs,^10–18^ water splitting catalysis,^19–27^ ammonia reaction catalysis,^28–35^ hydrocarbon reforming^36–48^ and CO_2_ electrolysis.^49–61^

The exsolution process was widely believed to be influenced by both internal and external factors. Intrinsic factors include defects, dopants, elemental composition, defect concentrations, strain, and facet effects, with defects such as A-site deficiency playing a pivotal role.^7,8^ A-site deficiency promotes the generation of oxygen vacancies, facilitating the exsolution of B-site cations^62,63^ and serve as active sites for nanoparticle nucleation.^64^ The host lattice and dopants influence the type, morphology, and population of exsolved nanoparticles, while strain and facets affect cation and oxygen vacancy formation, thus impacting exsolution behaviour.^8,9,65–67^ Among the extrinsic factors, reduction temperature is of paramount importance, and it can be readily managed. Tang *et al.*^68^ found that Rh cation exsolution from La_0.43_Ca_0.37_Rh_0.01_Ti_0.99_O_3_ preferred to nucleate at lower temperatures (500–700 °C), while particle growth occurred above 700 °C. Similarly, Cao *et al.*^69^ employed *in situ* transmission electron microscopy (TEM) to observe the exsolution process of Ni cations from LaNiO_3_ over time. Ni exsolution onset was noted at 600 °C with a preference for exsolution at grain boundary positions. Consequently, the exploration of external parameters such as reduction temperature is critical for understanding their impact on modulating the morphology and distribution of exsolved nanoparticles.

Double perovskite oxides, especially A_2_BB′O_6_, where A = rare earth, B, B′ = transition metal oxides, exhibits a distinctive crystal structure capable of hosting different elements at the B-site. This structural characteristic renders them highly applicable in SOCs. Yang *et al.*^70^ applied Co-substituted Sr_2_Fe_1.5_Mo_0.5_O_6−*δ*_ as the anode of SOFCs. Exsolution of Co metal nanoparticles greatly enhanced peak power density and the stability of anode materials, in which degradation was negligible for 190 hours in syngas and 300 hours in methane. Similarly, Wu *et al.*^71^ investigated the performance of the Sr_1.9_Fe_1.5_Mo_0.4_Cu_0.1_O_6−*δ*_ double perovskite anode with the exsolution of Fe and Cu phases, showing a great enhancement in power density at 800 °C of up to 1.2 W cm^−2^ in an electrolyte-supported SOFC. Yet, the exsolution phenomenon of metal nanoparticles from double perovskites and the influence of intrinsic and extrinsic factors still poses a challenge. Despite being a topic of interest, only a few studies have investigated this, for example, Du *et al.*^72^ studied Co–Fe exsolution from the Sr_2_FeMo_0.65_Co_0.35_O_6−*δ*_, proposing the similarities of lattice structure of the metal phase and perovskites reduced the interfacial energy and stabilized the exsolved nanoparticles, whereas the impact of external factors on morphology remains unexplored.

In our previous work,^73^ a low-temperature exsolution between 350–500 °C was observed in A-site deficient La_1.85_NiRuO_6−*δ*_ double perovskite samples, while the reduction temperature was found to have an impact on the particle size and distribution. However, the influence of the B-site cation ratio on this process remains insufficiently explored. To better separate the inherent B-site cation exsolution and the dopant exsolution, in this work, Ru-doped lanthanum nickel titanate double perovskites were adopted and their exsolution behaviour explored with reduction temperatures ranging from 350 °C to 1000 °C. The morphology of the materials reduced at high temperature (800 °C) and low temperature (450 °C) were studied, while *in situ* scanning transmission electron microscopy (STEM) coupled with energy dispersive X-ray (EDX) analysis was utilized to reveal the morphology changes of the double perovskite samples during the heating and reduction process. The electrochemical impedance spectra (EIS) reflecting the exsolution behaviour at different reduction temperatures and *I*–*V* curves of single SOCs were also studied, suggesting the potential of the nanoparticle decorated double perovskite as SOC electrodes.

## 2. Experimental method

### 2.1 Sample preparation

The La_2_NiTiO_6_, and La_2−*x*_NiTi_0.9_Ru_0.1_O_6−*δ*_ (*x* = 0, and 0.15) double perovskites, denoted as L2NT and L2-*x*NR respectively, were synthesized by the citrate–nitrate sol–gel method.^74^ Stoichiometric amounts of La(NO_3_)_3_·6H_2_O (Sigma-Aldrich, 99.999%), Ni(NO_3_)_2_·6H_2_O (Sigma-Aldrich, 99.999%) and Ru(NO)(NO_3_)_*x*_(OH)_*y*_ solution (Johnson Matthey, 99.8%) were mixed in citric acid solution (10 wt%, VWR chemicals). The solution underwent continuous stirring and was subjected to heating on a hotplate to facilitate evaporation before the start of the gelation process. Then TiO_2_ (Sigma-Aldrich, nano powder, 99.5%) was added into the solution to form homogeneous suspension. Given the limited solubility of the titanium source, continuous stirring was employed throughout the entirety of the gelation procedure to ensure the creation of a uniform dispersion of the titanium dioxide. The resulting gel was dried at 300 °C, followed by decomposition at 600 °C for over 12 hours, resulting in the formation of a grey precursor mixture. The resultant mixture was subjected to an initial calcination at 1200 °C for 12 hours in static air and then a second calcination at 1400 °C for 12 hours in static air, leading to the formation of the double perovskite product. After this, the double perovskite compounds were subjected to reduction to initiate exsolution using a humidified mixture of 5% H_2_/Ar gas (with approximately 3% water vapor content, achieved by passing the gas through a water bubbler) at predetermined temperatures, for a duration of 3 hours.

### 2.2 Cell fabrication

Dense pellets of La_0.8_Sr_0.2_Ga_0.8_Mg_0.2_O_3−*x*_ (LSGM) electrolyte were manufactured by pressing commercially sourced LSGM powder (Fuelcellmaterials, USA) into pellets with a diameter of 13 mm. These pellets were then subjected to sintering at a temperature of 1400 °C for a duration of 12 hours. Following this sintering process, the pellets were subjected to grinding and annealing procedures to ensure a clean flat surface with thickness approximately 4–500 μm to provide a robust support for electrode materials. The L1.85NTR electrode ink was prepared through ball-milling, vacuum drying and triple-roll milling. The synthesized L1.85NTR ceramic powder, which was dispersed in acetone, was placed into a high-energy ball mill to obtain a homogenous, fine ink powder. Then vacuum drying was adopted to ensure complete removal of any residual organic solvent. The product powder and ink vehicle (commercial ink vehicle, FCM R1835, Fuelcellmaterials Inc, USA) were manually mixed with a weight ratio of 2 : 1. Triple-roll milling was used to thoroughly disperse the electrode powder in the ink vehicle and eliminate aggregates. Screen printing of the electrode ink onto the electrolyte pellet was conducted by creating a mask of scotch tape with an 8 mm hole attached to the LSGM pellet surface, then painting a layer of electrode ink on to the surface using a steel blade to make sure a uniform layer was deposited. After drying the ink, the mask was removed, and the electrode layer was sintered on to the electrolyte at 1000 °C for two hours. Silver conductor paste, as a current collector (Sigma-Aldrich) was painted on to the surface of the electrode and annealed at 700 °C for two hours. The same ink deposition method was utilised to fabricate L1.85NTR fuel electrode on commercial half-cell SECC-2.0 with electrolyte-support configuration (Hionic™ Electrolyte/LSM-GDC, Fuelcellmaterials Inc, USA).

### 2.3 Characterization

X-ray diffraction (XRD) analysis was employed to examine the phase compositions of the samples both before and after reduction. This analysis was carried out using a PANalytical MPD diffractometer equipped with Cu Kα radiation, *λ* = 1.5406 Å. The crystal structure and lattice parameters of the double perovskites were obtained through Le Bail and Rietveld refinements, utilizing the FullProf software suite.^75^ To determine the cation ratio of the synthesized materials, inductively coupled plasma atomic emission spectroscopy (ICP-OES) was utilized using a Thermo Scientific iCAP 6000 Series ICP spectrometer. Microstructural evaluations of symmetric cells and the morphology of the double perovskite samples before and after reduction were conducted using a Zeiss Leo Gemini 1525 scanning electron microscope (SEM). Analysis of the acquired images was carried out using the ImageJ software (https://www.imagej.nih.gov) to derive nanoparticle sizes. Transmission electron microscopy (TEM) was employed to investigate the morphology and size of exsolved nanoparticles. A Thermo Fisher F200i microscope operating at 200 kV was utilized to capture bright-field (BF) and STEM dark-field (DF) images of the reduced sample. EDX elemental mapping was performed using the Thermo Fisher F200i microscope fitted with a Bruker X-flash EDX spectrometer and an ADF detector operating with a camera length of 98 mm at 200 kV. To prepare TEM samples, L1.85NTR powder was dispersed in isopropanol *via* ultrasonication for 20 minutes, and the resulting suspension was drop-cast onto holey carbon-coated copper TEM grids. *In situ* STEM studies were employed to explore the temperature and time dependence of exsolution phenomena. Pristine perovskite powder samples were loaded onto Protochips Fusion Select E-chips (E-FHDC-VO)^76,77^ which employ a silicon nitride membrane for support and heat conduction, along with holey carbon films as sample holders. The powder sample was diluted, suspended in isopropanol, and deposited onto the chips in a 3 μL suspension, left to dry in static air overnight. The sample and E-chip were then mounted onto a Protochips rod connected to an external power supply. The experiment involved heating the samples in vacuum conditions at a rate of 5 °C s^−1^ to the desired temperature and subsequently cooling at the same rate. Analysis of STEM images was conducted using Protochips Axon Studio software.

The electrochemical performance of the symmetrical cell (L1.85NTR/LSGM/L1.85NTR) was assessed through electrochemical impedance spectroscopy (EIS) to investigate exsolution processes at specific temperatures. A two-probe geometry was implemented using Pt mesh current collectors attached to opposite sides of the symmetrical cell. A frequency response analyser (Solartron Modulab XM ECS) was connected to the configuration, operating within a frequency range of 1 MHz to 50 mHz. The impedance spectra were measured while supplying 5% H_2_/Ar gas at a rate of 200 ml min^−1^ to the symmetrical cell. The Zview software (Version 3.5 by Scribner Associates, USA) was employed for the analysis of acquired EIS spectra. Full cell *I*–*V* curves and impedance spectra were obtained by placing the L1.85NTR/Hionic™ Electrolyte/LSM-GDC single cell in a rig based on an alumina tube sealed with ceramic sealant (AREMCO, lot No. 668-1263). Measurements were carried out at 850 °C with a thermocouple placed next to the sample to make sure thermal equilibrium was achieved. 50 ml min^−1^ hydrogen with 3% H_2_O was fed to the L1.85NTR electrode whilst the LSM-GDC electrode was kept in static air during the measurement. Similar two probe connections and EIS measurements were adopted, and a Solartron Modulab XM ECS was used to conduct the impedance measurements and DC controlled linear sweep voltammetry. Thermogravimetric analysis (Netzsch STA 449c F5) was utilized to understand material stability as a function of temperature under a flowing 5%H_2_/Ar atmosphere. After a correction run with an empty crucible, approximately 40 mg of the sample was loaded into a Pt crucible and heated to 1000 °C at a rate of 10 °C min^−1^. The change in mass was subsequently analysed using Netzsch Proteus software.

## 3. Results and discussion

### 3.1 Lattice structure and reduction of L1.85NTR

Building upon our prior investigation of ruthenate double perovskites,^73^ it was demonstrated that introducing a 7.5 mol% A-site deficiency significantly enhanced the exsolution of B-site cations. In the current study, we introduced A-site deficiency into Ru-doped lanthanum nickel titanium double perovskites. Stoichiometric lanthanum nickel titanate double perovskite, La_2_NiTiO_6_ and lanthanum nickel titanate double perovskite with 5 at% ruthenium dopant on the B-site, La_2_NiTi_0.9_Ru_0.1_O_6±*δ*_, denoted as L2NT and L2NTR, were synthesised as initial materials prior to the introduction of A-site deficiency within the structure. The X-ray diffraction (XRD) patterns and Le Bail refinement results are provided in Fig. S1 and Table S1 (ESI†). Both L2NT and Ru-doped L2NTR samples exhibited a monoclinic double perovskite structure with the space group *P*2_1_/*n*. Le Bail refinement was executed based on reference ICSD186433,^78^ showing slight lattice expansion with the 5 at% doping of ruthenium cations. ICP-OES results of the L2NT and L2NTR are shown in Table S2 (ESI†), indicating the intended chemical composition in the two phases.

**Fig. 1:**
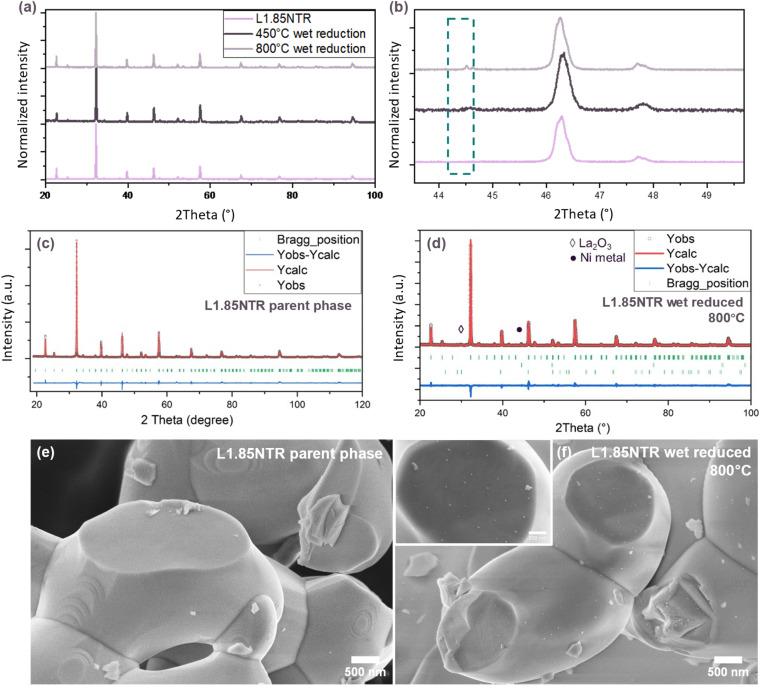
XRD patterns of the as-prepared L1.85NTR pristine, and L1.85NTR samples reduced at 450 °C and 800 °C in wet 5% H_2_/Ar (a) and zoomed in detailed region from 43° to 51° 2*θ* postion (b); (c) XRD pattern and Rietveld refinement plots of as-prepared L1.85NTR parent phase. The factors of agreement are *R*_p_ = 4.95, *R*_wp_ = 6.41, *R*_exp_ = 4.11 and *χ*^2^ = 2.43; (d) XRD pattern and Rietveld refinement profiles of L1.85NR reduced at 800 °C. The factors of agreement are *R*_p_ = 5.59, *R*_wp_ = 7.08, *R*_exp_ = 4.12 and *χ*^2^ = 2.95. Lattice parameters of nickel metal phase were refined based on reference structure ICSD64989.^79^ Lattice parameters of lanthanum oxide phase were refined based on reference structure ICSD28555;^80^ SEM images of (e) as prepared and (f) reduced L1.85NTR sample at 800 °C for 3 hours. The inset image is the enlarged display of the reduced sample.

Similarly, XRD and Rietveld refinement were employed to study the 7.5 at% A-site deficient phase, denoted as L1.85NTR. As with L2NT and L2NTR, space group *P*2_1_/*n* based on reference ICSD186433,^78^ was employed to analyse the XRD pattern of the A-site deficient double perovskite L1.85NTR using Rietveld refinement, as illustrated in Fig. 1c and S2 (ESI†). The green arrows indicate two subtle features in Fig. S2 (ESI†), which were attributed to Cu K_β_ diffraction peaks. These peaks arose due to the unavoidable incomplete filtering of the X-ray source and were excluded during the refinement fitting process. For Rietveld refinement, we incorporated constraints into the calculations of the XRD profiles, as detailed in the ESI. The outcomes of the Rietveld refinement are provided in Table S3 (ESI†). The refined lattice parameters were found to be *a* = 5.550(1) Å, *b* = 5.559(1) Å, *c* = 7.844(1) Å, and *β* = 90.046(1)°. The reduction in lattice dimensions and the increase in the *β* angle, as compared to the Le Bail refined data for the stoichiometric phase L2NTR, indicated that the introduction of A-site vacancies contributed to the contraction of the lattice volume. The ordering of B-site cations was quantified as *S* = 0.616 using eqn (S1) (ESI†), which suggested partial disordering of the B-site cations. Additionally, according to Table S3 (ESI†), the refined La cation occupancy exhibited a slightly higher value than the intended amount, implying a deficiency of 5.6 at%. ICP-OES results (Table S2, ESI†) showed a lower amount of A-site deficiency which might be attributed to unresolved phases depicted in Fig. S2 (ESI†) and the occurrence of local B-site enrichment.

Subsequently, the L1.85NTR sample underwent a reduction treatment in a wet 5% H_2_/Ar atmosphere. Various reduction temperatures were employed to assess the materials' stability under reducing conditions. The corresponding XRD patterns are presented in Fig. 1a and b, with additional details in Fig. S3 (ESI†). It can be inferred that the titanium-based sample maintained its double perovskite structure up to 1000 °C. However, a subtle peak at the 30° 2*θ* position indicated the presence of a small amount of La_2_O_3_ in the sample reduced at temperatures exceeding 800 °C (Fig. S3, ESI†). This observation is commonly linked to side reactions and exsolution, suggesting a slight decomposition of the double perovskite structure.^81,82^ Moreover, a peak at the 44.5° 2*θ* position was detected in the reduced samples, closely aligned with the Ni metal phase,^79^ signifying the occurrence of exsolution during the reduction process.

Rietveld refinement was applied to the XRD pattern of the L1.85NTR sample reduced at 800 °C (Fig. 1d). As illustrated in Fig. S4 (ESI†), subtle peaks attributed to a slight amount of La_2_O_3_ phase were observed at 29.96° and 29.05°. Therefore, both Ni and La_2_O_3_ phases were incorporated in the refinement, despite the less prominent peak intensity of La_2_O_3_ compared to the XRD patterns of L1.85NTR samples reduced at 900 °C and 1000 °C. The refined lattice and atomic parameters of the double perovskite phase reduced at 800 °C are outlined in Table S3 (ESI†). The lattice parameters of the double perovskite phase remained consistent with those of the as-prepared sample, while the atomic occupancies suggested a slightly greater B-site disordering and La-occupancy. This indicates a propensity to restore A-site stoichiometry through the exsolution process, as observed in previous studies.^6,8^ The refined lattice parameters of the Ni metal phase, 3.52395(7) Å, agreed with the reported reference ICSD 64989,^79^ confirming the exsolution of Ni. The refinement results revealed that the reduced sample contained 1.58(5) wt% Ni metal and 0.28(2) wt% lanthanum oxides. This finding implies that, under a reducing atmosphere, most of the A-site deficient sample experiences exsolution rather than decomposition into metal oxides.

Through the study of the sample's surface morphology after reduction at 800 °C in a wet 5% H_2_/Ar environment for 3 hours by SEM, nano-sized particles were observed evenly distributed on the surface of the oxide bulk, compared to the pristine sample (Fig. 1e and f). This suggested the occurrence of exsolution in the L1.85NTR sample during reduction.

### 3.2 Cation exsolution and temperature dependent morphology

**Fig. 2:**
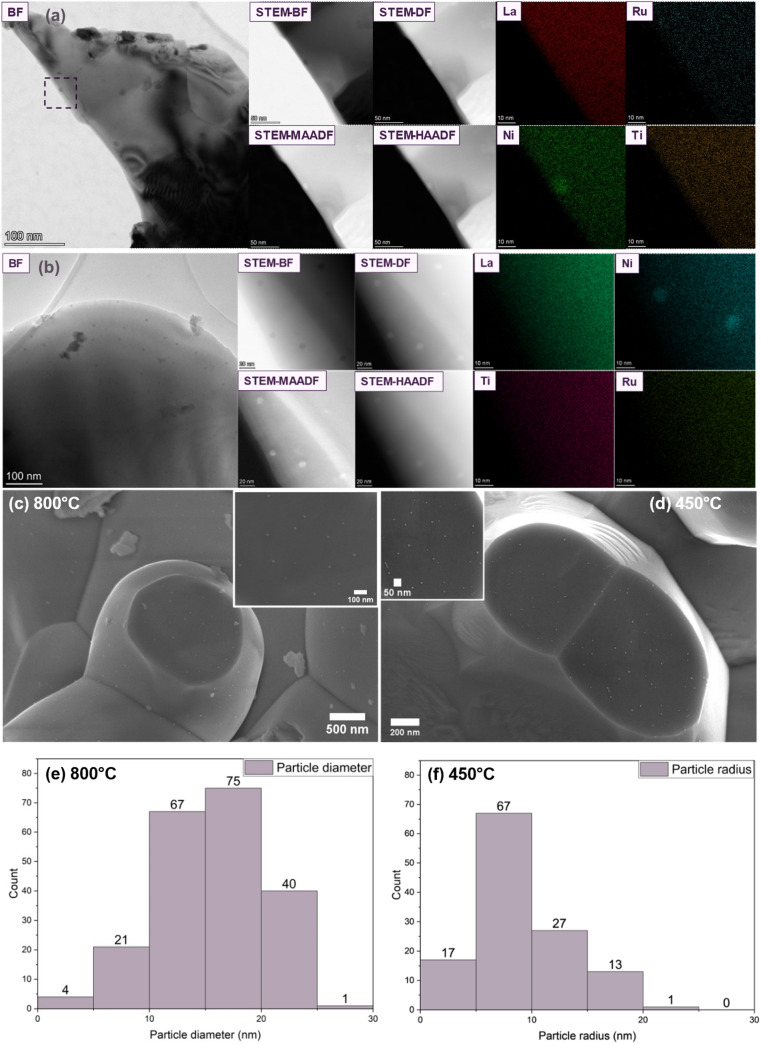
TEM bright field (BF) images and STEM annular bright field (ABF), annular dark field (ADF), medium angle annular dark field (MAADF) and high angle annular dark field (HAADF) images of L1.85NTR sample reduced at 800 °C (a) ans 450 °C (b) in wet 5% H_2_/Ar for 3 hours. The corresponding elemental distribution maps of La, Ni, Ru and Ti are listed at the left of the STEM images; SEM images of L1.85NTR sample reduced at 800 °C (c) and 450 °C (d) for the particle analysis and corresponding size distribution diagrams (e and f). The inset images in (c and d) displayed the details of the exsolved nanoparticles, which were analysed for particle size and size distribution.

Lower temperature reductions were also investigated, and the results were characterized through XRD, SEM, TEM, and STEM-EDX analyses. As depicted in Fig. 1b, the L1.85NTR sample reduced at 450 °C for 3 hours in a wet 5% H_2_/Ar environment exhibited faint diffraction signals of the Ni metal phase at 44.5°. In Fig. 2a and b, the bright-field TEM images, along with corresponding STEM images and EDX maps, are presented for L1.85NTR reduced at both 800 °C and 450 °C for 3 hours in wet 5% H_2_/Ar. Notably, for the sample reduced at the lower temperature (450 °C), STEM images revealed an abundance of exsolved nanoparticles. In line with the XRD and Rietveld refinement data for both samples, the EDX maps showcased the exsolved nanoparticles as a Ni metal phase. On the other hand, no significant enrichment of the other three elements was observed, underscoring the non-exsolution of Ru and Ti under the present conditions. This observation aligns with previous findings on titanate materials,^6,8^ where Ti cations tend to remain in the perovskite structure during the exsolution process due to their strong bonds with oxygen anions and low reducibility. Consequently, the presence of Ti cations at the B-site positions enhances the stability of the B-site ordered double perovskite structure during the reduction and exsolution processes. Compared with the ruthenate samples reported previously,^73^ this highlights the enhanced structural stability conferred by Ti cations.

To gain insight into the exsolution process at varying reduction temperatures, SEM images of the L1.85NTR sample reduced at 800 °C and 450 °C (as depicted in Fig. S5 and S6, ESI†) were analysed using ImageJ software.^83^ Specifically, the inset images in Fig. 2c and d display one of the selected areas for particle size analysis, deliberately excluding large grain fragments. The outcomes of particle size distribution analysis are presented in Fig. 2e and f. As anticipated, the sample reduced at the higher temperature (800 °C) exhibited larger particle sizes. In total, 208 particles from four areas (4.9 × 104 nm^2^) of the sample reduced at 800 °C were analysed, with particle density of 420 μm^−2^. The average size of the exsolved nanoparticles in the L1.85NTR sample reduced at 800 °C was approximately 15.8 nm, corroborating findings from TEM and STEM imaging. The majority of the sizes of exsolved nanoparticles following reduction in a wet 5% H_2_/Ar environment for 3 hours fell within the range of 10 to 20 nm. In contrast, the sample reduced at 450 °C exhibited smaller exsolved nanoparticles with higher particle density (1300 μm^−2^) in comparison to the samples reduced at 800 °C. A total of 125 particles from four areas covering an area of 9.6 × 10^3^ nm^2^ were analysed, yielding an average size of approximately 8.7 nm. The size distribution in Fig. 2f reveals that 67 out of the 125 particles measured between 5 to 10 nm, displaying a relatively narrower distribution than observed in the L1.85NTR sample reduced at 800 °C. This observation suggests a more uniform morphology for the exsolved nanoparticles at the lower temperature. The increasing particle size and broadened size distribution from 450 °C to 800 °C implies particle-growth dominated exsolution in this temperature range.

**Fig. 3:**
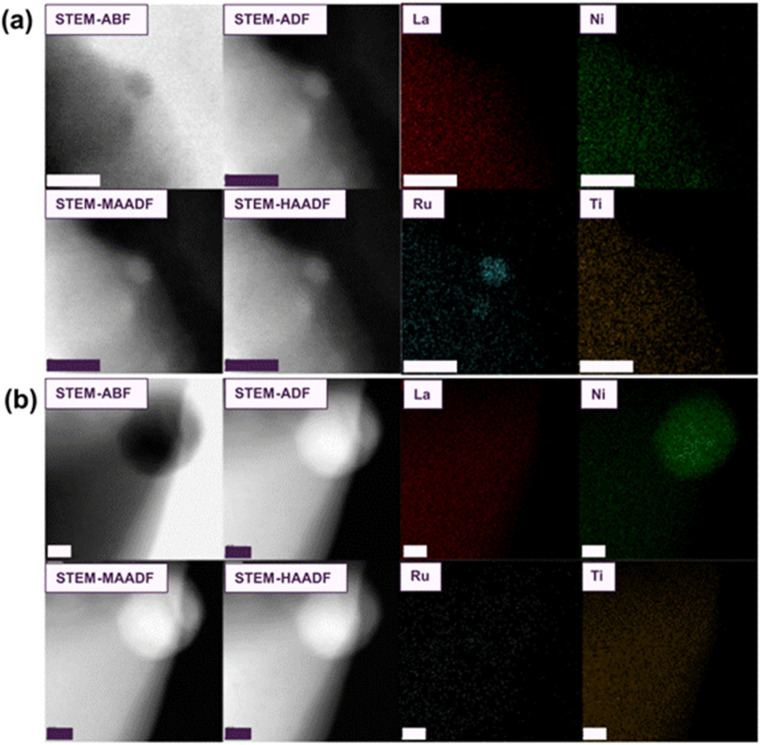
STEM images and EDX maps of two distinct regions (a) and (b) of the L1.85NTR sample reduced at 1000 °C in a wet 5% H_2_/Ar for 3 hours. Scale bar: (a) 5 nm; (b) 10 nm.

To further investigate the phenomenon of Ru-exsolution, STEM-EDX examination was conducted on the L1.85NTR sample that underwent reduction above 800 °C in a wet 5% H_2_/Ar environment. Fig. 3 displays the STEM-EDX analysis performed on two distinct particles situated on the surface of the L1.85NTR sample, which was reduced at 1000 °C for 3 hours in the same wet 5% H_2_/Ar atmosphere. Fig. 3a prominently shows the occurrence of Ru exsolution, manifesting as particles with sizes ranging from approximately 2.5 to 3 nm. Notably, there was an absence of Ni enrichment within these exsolved nanoparticles. In contrast, Fig. 3b offers a depiction of Ni particle exsolution, with these particles measuring around 35 nm in size. These observations highlight the distinctive characteristics of ruthenium exsolution within lanthanum nickel titanates from nickel exsolution. Specifically, the exsolution process of Ru cations demands higher reduction temperatures. This behaviour can be attributed to the gradual migration of transition metal cations, particularly Ru cations, within the perovskite structure.^84^ This sluggish diffusion contrasts to the behaviour observed in ruthenates,^73^ where Ru cations were more widely distributed within the material. This indicates the need for elevated temperatures, such as 1000 °C in a wet 5% H_2_/Ar environment, to promote the diffusion/exsolution of Ru cations from the interior of the parent L1.85NTR perovskite structure to its surface.

### 3.3 *In situ* observation of Ni cation exsolution

**Fig. 4:**
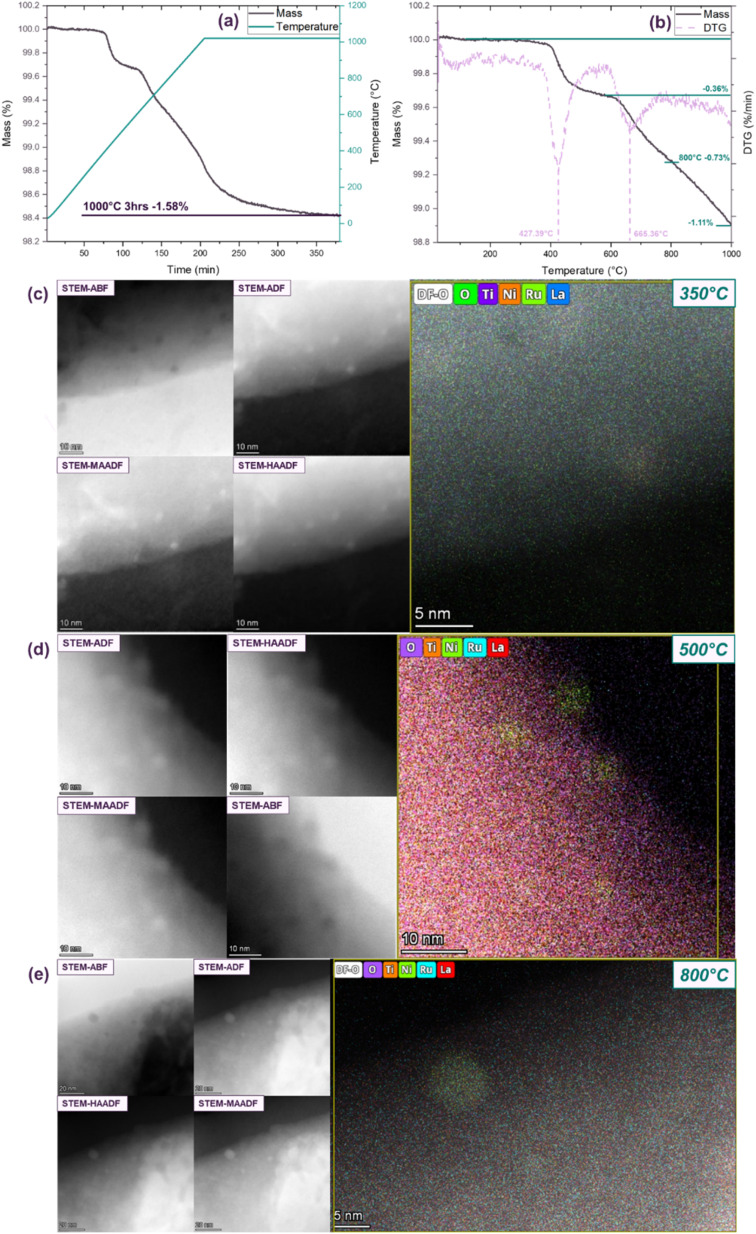
(a) TG curves and corresponding temperature profiles as a function of time, encompassing both the heating and dwelling phases; (b) TG and DTG curves as a function of increasing temperature, ranging from room temperature (RT) to 1000 °C. (c) STEM-ABF, STEM-ADF, STEM-MAADF, STEM-HAADF images and maps of the quenched L1.85NTR after reduction at 350 °C. Same area with the *in situ* observation in Fig. S8.† STEM-ABF, STEM-ADF, STEM-MAADF, STEM-HAADF images and EDX maps of different areas in the quenched L1.85NTR after reduction at 500 °C (d) and 800° (e).

To further explore the dependence of exsolution behaviour on reduction temperature, we employed *in situ* characterization techniques including thermogravimetric analysis (TGA) and STEM to examine the L1.85NTR sample. Fig. 4a and b illustrates the TGA results and its first derivative (DTG) for the L1.85NTR sample. Following reduction at 1000 °C for 3 hours in dry 5% H_2_/Ar, the mass loss approached a plateau, illustrating a 1.58 wt% weight loss. To clarify the differences between the dry and wet gases, mass spectrometer analysis was utilized in this work and are detailed in Table S5 (ESI†). Additionally, to validate the effect of A-site deficiency on lanthanum nickel titanate exsolution, the TGA plot of the stoichiometric phase L2NTR is shown in Fig. S7 (ESI†). Similar mass loss behaviour to L1.85NTR was observed, albeit with a substantially smaller mass loss (0.45 wt%), underscoring the significance of the 7.5 at% A-site deficiency in promoting exsolution.

Prior to reaching 1000 °C, two distinct phases of mass loss became evident as illustrated in Fig. 4a and b. The mass loss attributed to surface-adsorbed moisture was negligible, as indicated by the absence of noticeable weight change within the temperature range of room temperature to 250 °C.^85–87^ The DTG curve indicated that the first peak of maximum mass loss occurred around 427 °C, followed by a broader mass loss phase centred at approximately 665 °C. The exsolution of Ni nanoparticles was confirmed through the characterization of the L1.85NTR sample reduced at 450 °C and 800 °C. Both mass loss steps were linked to the exsolution process, resulting from the loss of oxygen anions in the perovskite structure.^88,89^ As the temperature increased, the first plateau emerged at around 500 °C, accompanied by a 0.36 wt% mass loss. Subsequently, when the temperature surpassed 600 °C, a continuous mass loss was observed, akin to the reported exsolution behaviour of La(Sr)Cr_0.85_Ni_0.15_O_3−*δ*_ (ref. 90) and La_0.6_Sr_0.4_FeO_3−*d*_.^86^ The loss of oxygen was deduced from the TG curve, as depicted in eqn (S2) (ESI),† revealing an oxygen loss of approximately 0.25 mol per mol of L1.85NTR for the sample reduced at 800 °C. Consequently, in conjunction with the outcomes of Rietveld refinement (Fig. 1d) and Table S4 (ESI†), the reduction process at 800 °C can be summarized as follows:
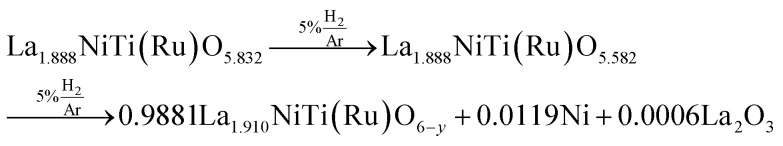


However, precisely determining the oxygen stoichiometry of the resulting La_1.910_NiTi_0.9_Ru_0.1_O_6−*y*_ double perovskite remains challenging due to the uncertainty surrounding the exsolution behaviour of Ni cations or Ni–O species.

*Ex situ* microscopy characterization disclosed that exsolution occurred not only at high temperatures but also at relatively lower temperatures. Thermogravimetric analysis indicated two stages of mass loss, signifying two distinct exsolution stages. Thus, *in situ* STEM was employed to examine the evolution of the sample's morphology. The Protochips Fusion Select E-chips and rod were utilized to attain the elevated temperatures necessary for the exsolution process. In contrast to the experimental conditions, high vacuum (∼10^−7^ torr, equivalent to ∼1.33 × 10^−5^ Pascal) was employed as the reducing environment in the TEM equipment. To thoroughly investigate the evolution of L1.85NTR during the exsolution process, a heating procedure was employed, involving gradual temperature increments to minimize vibrations caused by rapid heating of the sample and rod. The thermal cycle applied to the sample is outlined in Fig. S8 (ESI†). Additionally, to analyse the chemical composition of the exsolved nanoparticles during thermal treatment, STEM-EDX analysis was conducted after swiftly quenching the sample to room temperature. Both heating and quenching rates were set at 5 °C s^−1^.

In alignment with the *ex situ* characterization, the STEM-EDX analysis of the sample, quenched between heating stages, unveiled the presence of exsolved Ni nanoparticles, along with a progressive enlargement in particle size (as illustrated in Fig. 4c–e). As depicted in Fig. 4c, the dot contrasts situated at the edge of the bulk grain exhibited measurements of approximately 3–3.5 nm. Fig. 4d presented STEM images and EDX maps of exsolved nanoparticles quenched from 500 °C, showcasing particles ranging in size from 3.1 nm to 5.5 nm. Upon reaching 800 °C, fully developed spherical nanoparticles with socket structures became evident. The spherical particle depicted in Fig. 4e had an approximate diameter of 9 nm. Notably, since the *in situ* growth of these nanoparticles took place under vacuum conditions rather than in a hydrogen atmosphere, the measured diameters of the Ni particles appeared to be slightly smaller than those depicted in Fig. 2c–f.

An intriguing observation of morphology reconstruction was observed during the *in situ* reduction of L1.85NTR (depicted in Fig. S9, ESI†), showing STEM-ADF images of the initial sample and the sample at various temperatures: 350 °C, 450 °C, and 800 °C. At a reduction temperature of 350 °C, three distinct bright dots, marked by arrows, are discernible on the surface of the sample grain, diverging from the initial pristine sample. These particles in this region are exactly those shown in Fig. 4c, corroborating their Ni metal composition in line with the *ex situ* observations. As illustrated in Fig. S9b,† when the temperature reaches 450 °C, nanoparticles indicated by the green arrows reincorporate or dissolve into the bulk, vanishing from microscopic observation. This phenomenon indicates a reconstruction of both the sample's surface morphology and the exsolved nanoparticles, concomitant with mass movement within the bulk oxide materials. The nanoparticles nucleation and growth on the surface of ceramic oxides involves the interplay of segregation energy, surface energy and lattice mismatching on each facet.^9,64–66,91^ This reconstruction likely arises from exsolution as a dynamic process, continuously modifying the surface morphology until the exsolved nanoclusters/nanoparticles achieve both an energetically favourable state and a critical size for further growth. In a related study, Cao *et al.*^64^ computed the theoretical critical size of Ni nanocrystals to be approximately 1.5 nm. In our specific case, STEM-EDX analysis (Fig. 4c) demonstrated nanoparticle sizes of 3–3.5 nm, equivalent to 12–15 atomic diameters.

**Fig. 5:**
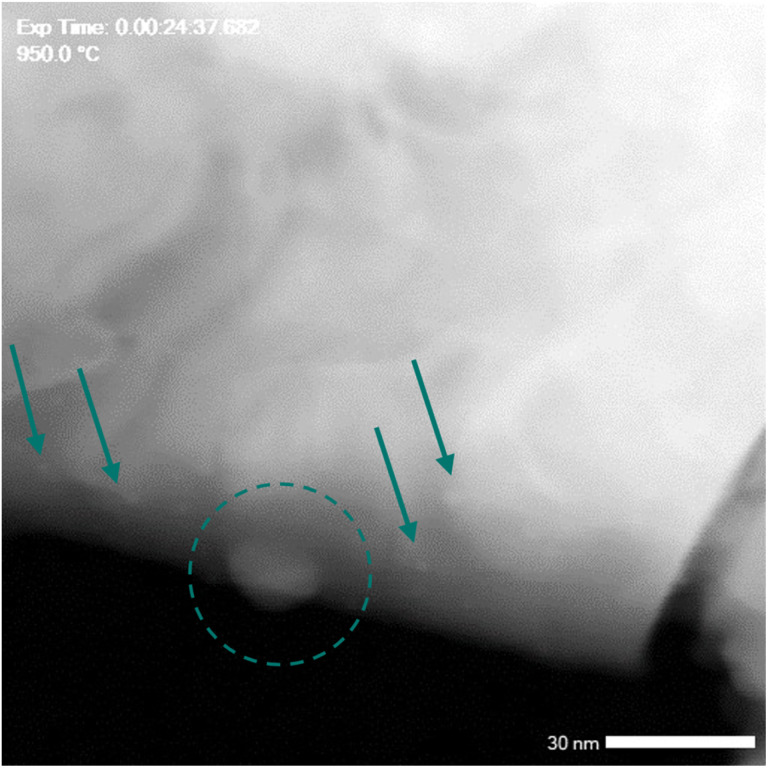
*In situ* STEM-ADF image of the L1.85NTR sample measured at 950 °C in high vacuum. The green arrows highlight the exsolved nanoparticles/nuclei with smaller size.

Another facet observed under *in situ* conditions, providing additional evidence for the nucleation and growth of particles, is illustrated in Fig. 5. Two types of nanoparticles were evident on the surface of the bulk grain reduced at 950 °C. One type comprised small nanoparticles, akin to those seen in Fig. 4c–e and S9 (ESI†), with sizes of around 3 nm, equivalent to 12 atomic diameters. The other type, highlighted by the green circle in Fig. 5, exhibited larger particle sizes of about 17 nm. The larger particles were indicative of particle growth, while the smaller particles might remain in a metastable state, potentially rejoining the bulk or merging with neighbouring larger particles through Ostwald ripening as the reduction temperature/time are extended.^64,92^ The nucleation and reconstruction of exsolved Ni nanoparticles offer insights into the two-step exsolution process depicted in Fig. 4a and b, reflecting the TG behaviour of L1.85NTR during reduction.

### 3.4 *In situ* EIS and electrochemical performance

**Fig. 6:**
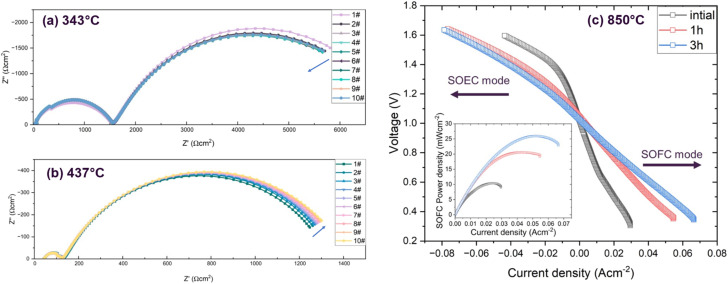
EIS spectra of L1.85NTR/LSGM/L1.85NTR symmetric cell measured in flowing wet 5% Ar at 343 °C (a) and 437 °C (b). The spectra of each temperature were measured continuously with the reduction progressing. Each spectrum was scanned for 30 min. (c) *I*–*V* curves of the L1.85NTR/Hionic™ Electrolyte/LSM-GDC single cell tested at the initial stage of reduction, 1 h and 3 h after reduction in wet H_2_ (3% H_2_O). The inserted plots shows the powder density of the SOFC mode.

To assess the electrochemical performance of the L1.85NTR sample and its temperature-dependent behaviour, impedance spectra of symmetric cells employing a L1.85NTR/LSGM/L1.85NTR configuration were measured. These measurements were conducted at open-circuit voltage and specific temperatures within a wet 5% H_2_/Ar atmosphere. A detailed depiction of the measurement setup and the microstructure of the symmetric cells can be found in Fig. S11 (ESI†). Impedance spectra were captured at temperatures of 343 °C, 437 °C, 528 °C, and 710 °C (Fig. 6 and S10, ESI†). Each measurement consisted of 10 cycles of continuous data acquisition. Additionally, EIS spectra at 800 °C were recorded after 4 hours of reduction in a wet 5% H_2_/Ar atmosphere. The scanning procedure encompassed traversing from high frequency to low frequency, with each spectrum necessitating approximately 30 minutes to be fully scanned. This approach facilitated the observation of spectral evolution as the reduction process unfolded within the specified temperature range of interest.

Fig. 6a reveals two intricate arcs spanning from high to low frequencies, as depicted in the spectra recorded at 343 °C. The gradual progression observed in the ten spectra over a span of five hours implies a subtle decline in impedance as the exsolution process unfolds. This trend seems to stabilize after 30 minutes of measurement at this temperature. Interestingly, an unexpected phenomenon was observed as the temperature increased to 437 °C: during the reduction process, the low-frequency impedance arc exhibited a slight increase (Fig. 6b). Upon further elevating the temperature beyond 500 °C, the impedance arcs demonstrated a more pronounced reduction, as evident in Fig. S10a and b (ESI†). In contrast to the observations in Fig. 6a, the spectra obtained at 528 °C and 710 °C indicated that the exsolution persisted for a longer duration. The spectra acquired at 528 °C appeared to stabilize after 4 hours of reduction, while the spectra captured at 710 °C (depicted in Fig. S10b, ESI†) continued to decrease until the reduction was extended to 5 hours. This suggested that the limited amount of Ni cations located at the B-site were exsolved at lower temperature and is highly dependent on the reduction temperature.

Two R/CPE (resistor and constant phase element) components were employed to simulate the frequency-dependent impedance responses. This was performed on the symmetric cell response after reduction at 800 °C for 4 hours (Fig. S10c, ESI†). The details of this analysis can be found in Table S6 (ESI†). Specifically, the low-frequency arc was associated with the electrode–electrolyte interface, implying an enhancement in conductivity at this interface, likely stemming from the advancement of Ni cation exsolution. In light of the earlier *in situ* observations conducted using TEM and TGA, an unusual behaviour of the L1.85NTR electrode was identified within a relatively low temperature range, approximately 400–450 °C. This phenomenon could potentially account for the observed rise in impedance at 437 °C. The reconstruction of nanoparticles, accompanied by the incorporation of Ni into the structure, alongside the loss of smaller nanoparticles, might impede the desorption of hydrogen and consequently lead to increased polarization resistance within this temperature range.

Full cell testing was conducted to evaluate the electrochemical performance of the L1.85NTR as a fuel electrode in SOCs and to assess the impact of Ni nanoparticle exsolution. Fig. 6c displays the *I*–*V* curve of the single cell L1.85NTR/Hionic™ Electrolyte/LSM-GDC measured at 850 °C. Consistent with the *in situ* EIS measurements, decrease of the impedance of a single cell confirmed the progression of Ni nanoparticle exsolution (Fig. S12, ESI†). After a 30 minute reduction, the impedance of the single cell decreased from approximately 37.3 Ω cm^2^ to 11.3 Ω cm^2^, indicating a reduction in the polarization resistance due to the exsolution of Ni nanoparticles. Compared to the impedance spectrum of symmetric cells measured at 800 °C (Fig. S10c, ESI,† the real axis intercept of the single cell spectra is smaller. This is primarily attributed to the lower resistance of the thin Hionic™ electrolyte at 850 °C. However, the single cell after reduction in wet hydrogen for 30 minutes exhibited higher polarization resistance compared to the spectrum of the symmetric cell (Fig. S10c, ESI†). This higher polarization resistance is likely due to complex reactions involving higher activation resistance of the cathode-side oxygen reduction reaction, interfacial resistance at the LSM-GDC/electrolyte interface, contributions from the LSM-GDC cathode resistance, and gas diffusion polarization. Following a 3 hour reduction of the L1.85NTR fuel electrode, the current density increased from 27 mA cm^−2^ at an applied voltage of 1.5 V to 59 mA cm^−2^, indicating an enhancement in the performance of the cell in SOEC mode. For SOFC mode, the peak power densities doubled after 3 hours of reduction and exsolution of the Ni nanoparticles (from 10 mW cm^−2^ to 25 mW cm^−2^). Whilst this is a low overall cell performance these data underscore the significant potential of metal nanoparticles in the electrode for improving the performance of both SOFCs and SOECs.

## 4. Conclusion

In this study, double perovskite materials comprised of Ru-doped lanthanum nickel titanates, were synthesized using the sol–gel method. To facilitate the exsolution process, A-site deficiency was introduced. XRD and Rietveld refinement analysis confirmed the monoclinic *P*2_1_/*n* structure of A-site deficient Ru-doped lanthanum nickel titanates (L1.85NTR), resembling stoichiometric phase L2NTR and undoped lanthanum nickel titanate L2NT. Upon reduction in a wet 5% H_2_/Ar environment, XRD patterns revealed retention of the double perovskite structure and presence of Ni metal phase. SEM and TEM images showed a uniform distribution of Ni nanoparticles over the surface of the reduced sample. Temperature-dependent exsolution process were comprehensively investigated through *ex situ* and *in situ* characterizations. SEM images show a smaller particle size and more uniform distribution of exsolved Ni nanoparticles after reduction at 450 °C compared to reduction at 800 °C. An intriguing exsolution phenomenon was observed within the temperature range of 350–500 °C, as evidenced by *in situ* TGA and microscopic observations. An unstable nucleation and reconstruction on the double perovskite surface was observed at this temperature range. EIS measurements on symmetric cells with L1.85NTR electrodes demonstrated impedance decrease with exsolution progression, while spectra at 437 °C showed increased low-frequency arc, attributed to electrode–electrolyte interface changes. The *I*–*V* curves of L1.85NTR/Hionic™ Electrolyte/LSM-GDC single cell exhibited enhanced performance after 3 hour L1.85NTR electrode reduction. Peak power densities in SOFC mode doubled from 10 mW cm^−2^ to 25 mW cm^−2^ after Ni nanoparticles exsolution. Limited reduction up to 1000 °C was observed due to low Ru cation content and diffusivity. These findings offer compelling evidence of our ability to finely adjust both the intrinsic exsolution of B-site cations and the dopant-induced exsolution within titanium-based double perovskites. Notably, we observed a significant reduction in impedance and a twofold increase in power density in single cells utilizing L1.85NTR as the fuel electrode at a temperature of 850 °C. These results underscore the promising potential of using nickel nanoparticle-decorated double perovskite electrodes in SOCs.

## Data availability

The data supporting this article can be found at Zenodo (https://www.zenodo.org/communities/electroceramic-materials/). These data include X-ray diffraction, electron microscopy and electrochemical impedance spectroscopy data. Further data are also included as ESI† to this article.

## Conflicts of interest

There are no conflicts to declare.

## Supplementary Material

NA-006-D4NA00349G-s001
