# Supplementary material for: In situ investigation of ruthenium doped lanthanum nickel titanium double perovskite and its exsolution behaviour
Source: Nanoscale Adv. 2024 Jul 8;6(17):4394–406. doi: 10.1039/d4na00349g (PMC11334975; doi:10.1039/d4na00349g)
Supplement: NA-006-D4NA00349G-s001 [file NA-006-D4NA00349G-s001.pdf]

Electronic Supplementary Material (ESI) for Nanoscale Advances.
This journal is © The Royal Society of Chemistry 2024

In-situ investigation of ruthenium doped lanthanum nickel titanium double perovskite and its exsolution behaviour

*Jia Guo[1], Andrey Berenov[1], Stephen J. Skinner[1,2]**

1 Department of Materials, Imperial College London, Exhibition Road, London, SW7 2AZ, UK
2 International Institute for Carbon Neutral Energy Research, Kyushu University, Fukuoka, Japan
E-mail: s.skinner@imperial.ac.uk

1. Figures

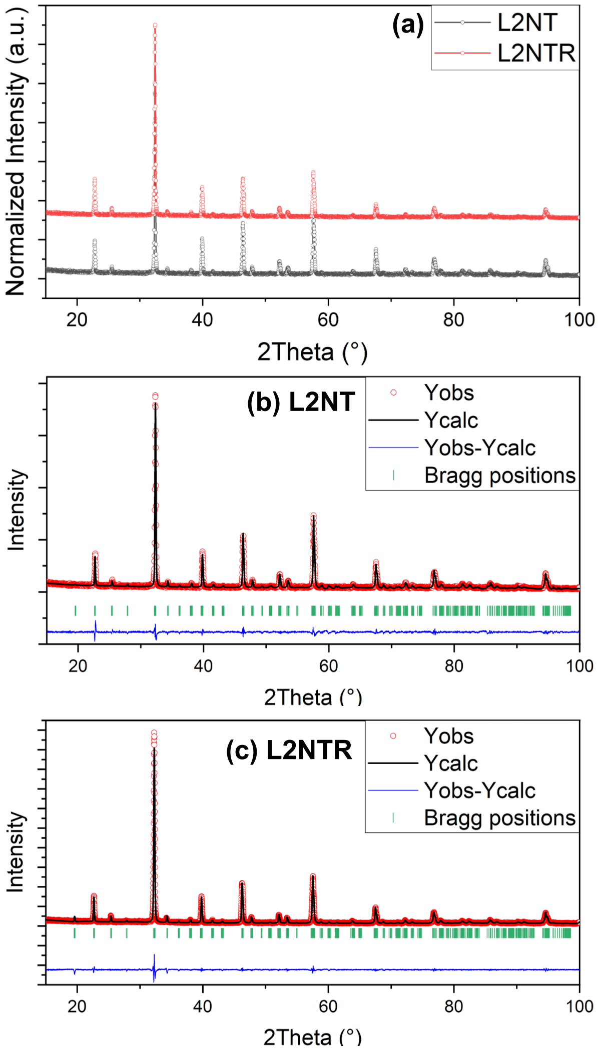

Figure S.1 (a) XRD patterns of stoichiometric lanthanum nickel titanate, L2NT, and Ru-doped lanthanum nickel titanate, L2NTR; (b) Le Bail refinement profiles of L2NT with R_p = 6.87, R_{wp} = 10.3, R_{exp} = 6.24 and χ^2= 2.71; (c) Le Bail refinement profiles of L2NT with R_p = 7.00, R_{wp} = 11.3, R_{exp} = 6.27 and χ^2= 3.23.

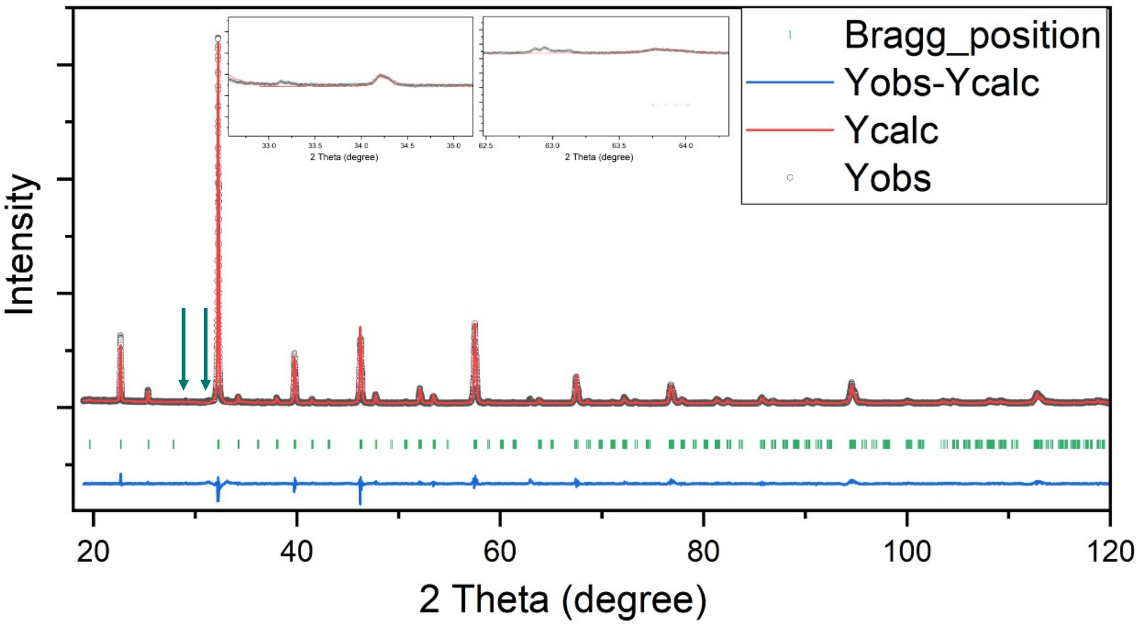

Figure S.2 XRD pattern and the Rietveld refinement profiles of L1.85NTR. The factors of agreement are $R_p = 4.95$, $R_{wp} = 6.41$, $R_{exp} = 4.11$ and $\chi^2 = 2.43$. Two inset plots are the zoomed pattens of unresolved peaks. The green arrows mark the Cu K_β reflections. Two subtle features were located at 33.2° and 62.5°, remaining unresolved. However, given the main diffraction signals strongly correspond to lanthanum nickel titanate double perovskite, the product was considered as majority single phase and adopted in the following study.

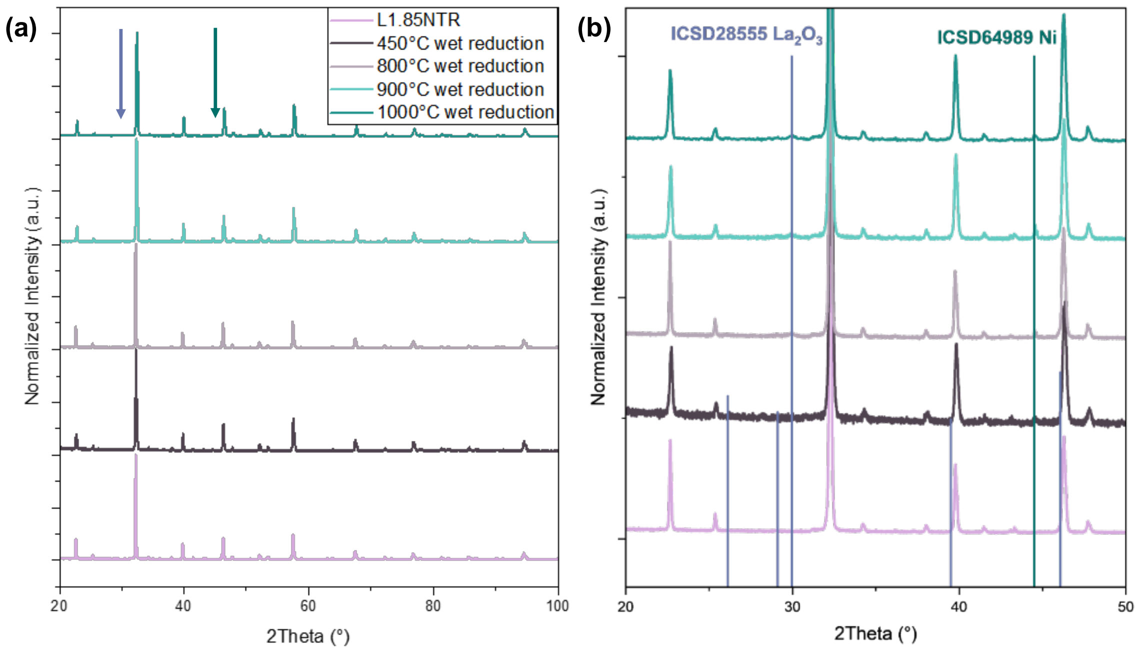

Figure S.3 (a) XRD patterns of A-site deficient L1.85NTR as prepared and reduced at 450°C, 800°C, 900°C and 1000°C. (b) zoomed-in XRD profiles at 20°-50° 2q range. The droplines indicate the Bragg position of La_2O_3 phase (ICSD28555[1]) and nickel metal phase (ICSD64989[2]).

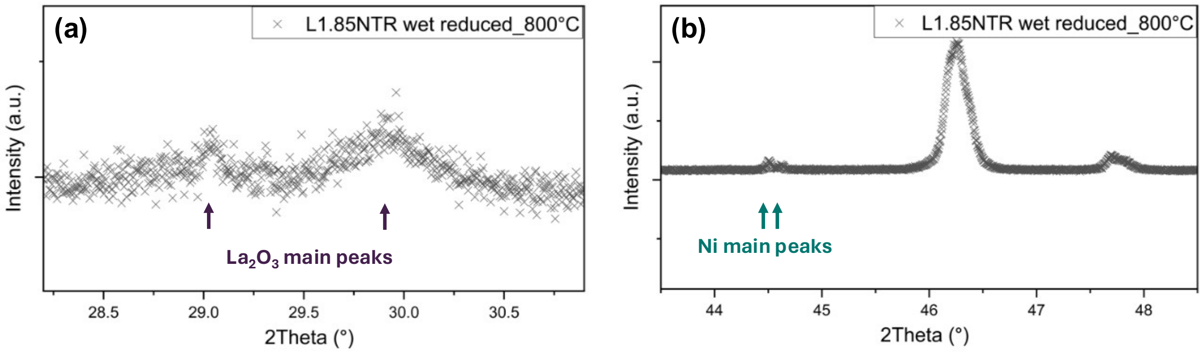

Figure S. 4 XRD patterns of L1.85NTR sample reduced at 800°, with 2 theta positions ranging from 28.2° to 30.9° highlighting the main peaks of La_2O_3 (a), and from 43.5° to 48.5° highlighting the main peaks of Ni metal phase (b).

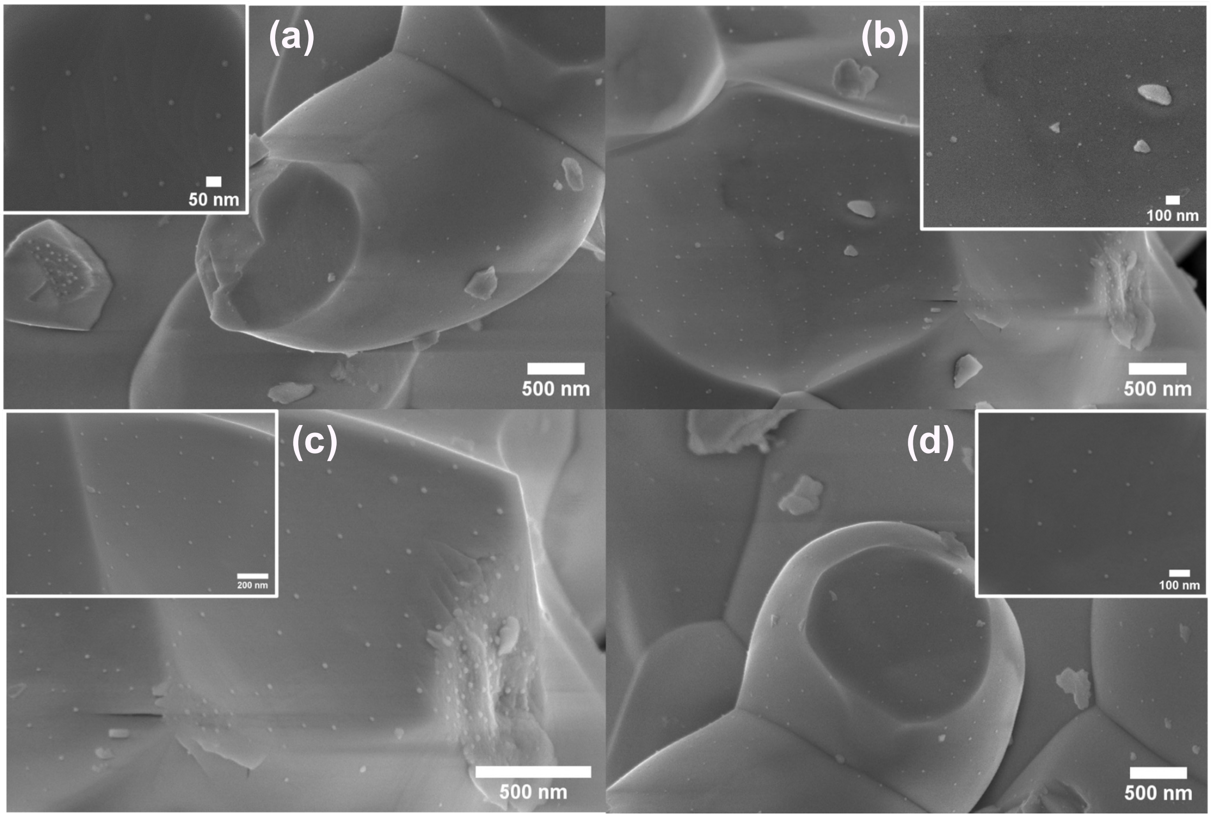

Figure S.5 SEM images of L1.85NTR sample reduced at 800°C for the particle analysis.

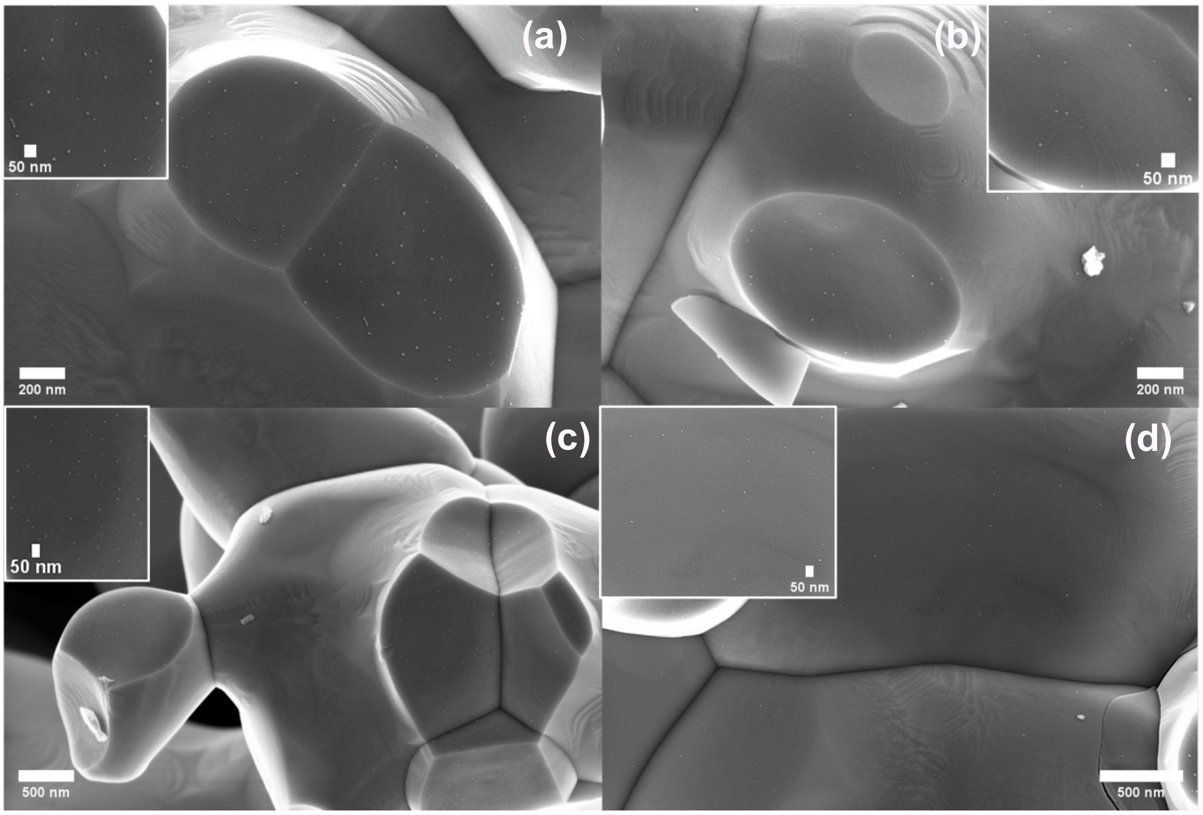

Figure S. 6 Size distribution diagrams of exsolved nanoparticles of L1.85NTR reduced in wet 5%H_2/Ar at 800°C(a) and 450°C(b) for 3 hours.

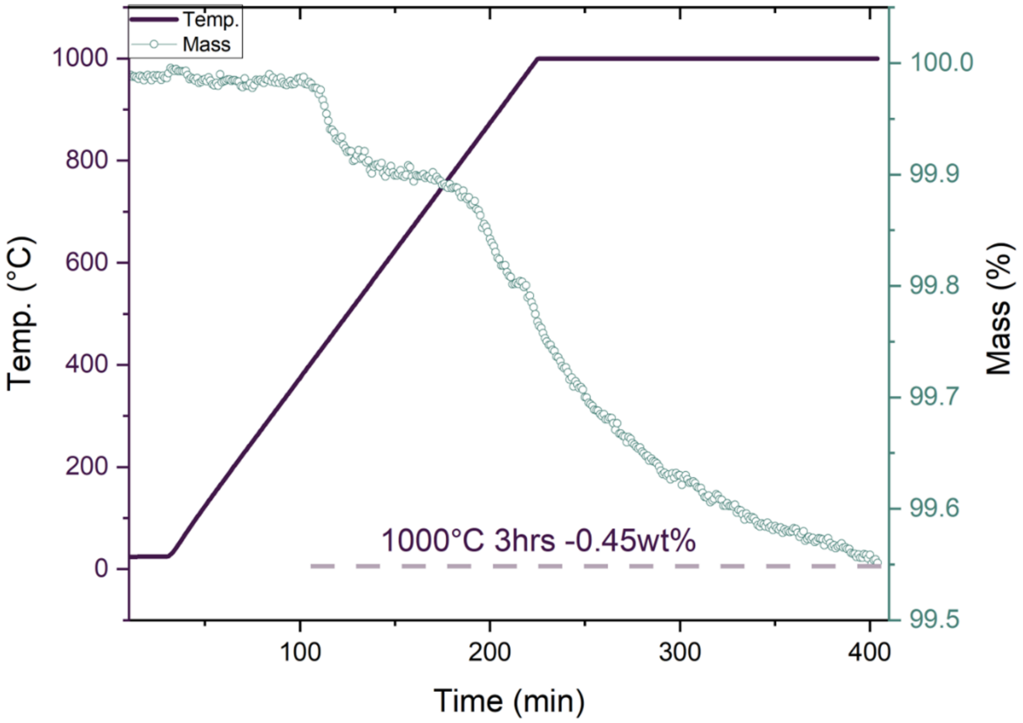

Figure S. 7 TG curve of stoichiometric sample L2NTR and corresponding temperature profile as a function of time in 5%H_2 atmosphere. The mass loss behaviour exhibited a two-step mass loss,

consistent with that observed in the A-site deficient sample L1.85NTR. However, a significantly smaller mass loss was noted, indicating a limited extent of reduction and exsolution in the stoichiometric phase.

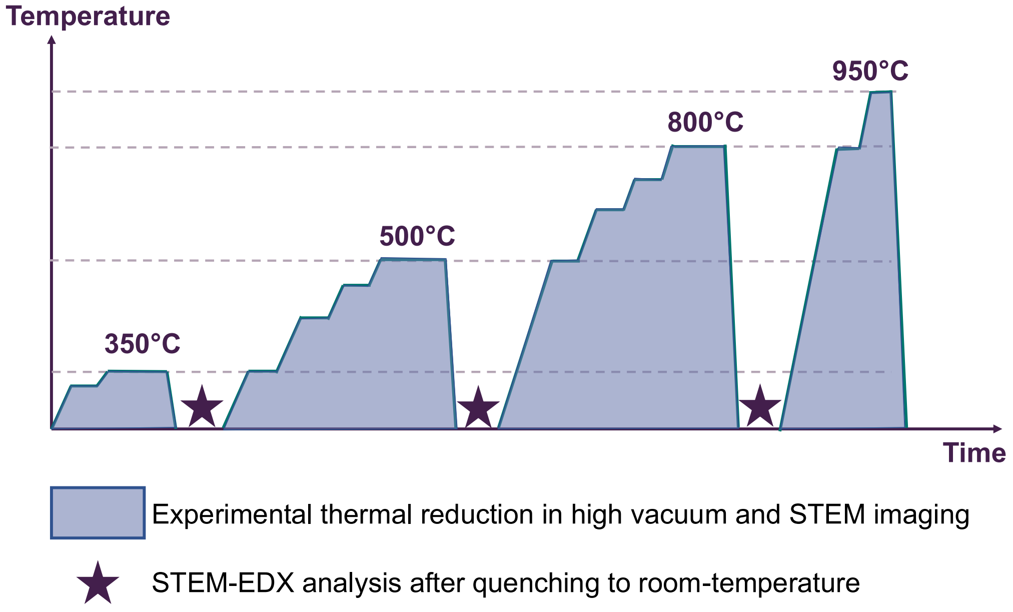

Figure S.8 Schematic illustration of the thermal cycle applied to the samples.

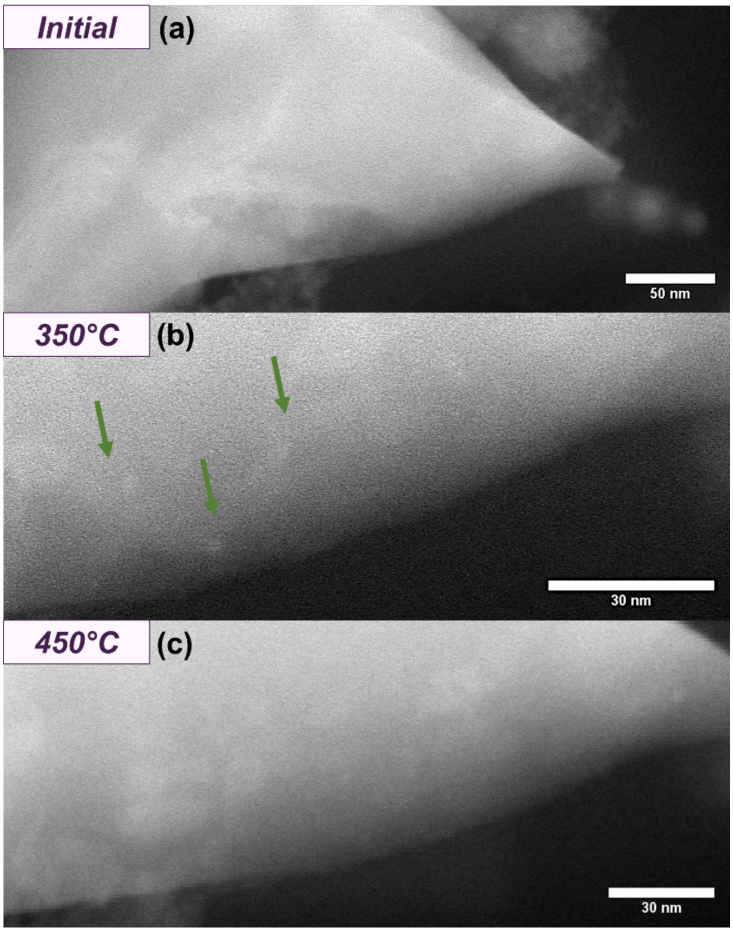

Figure S.9 *In-situ* STEM-ADF images of (a) initial and reduced L1.85NTR sample at 350°C (b) and 450°C (c).

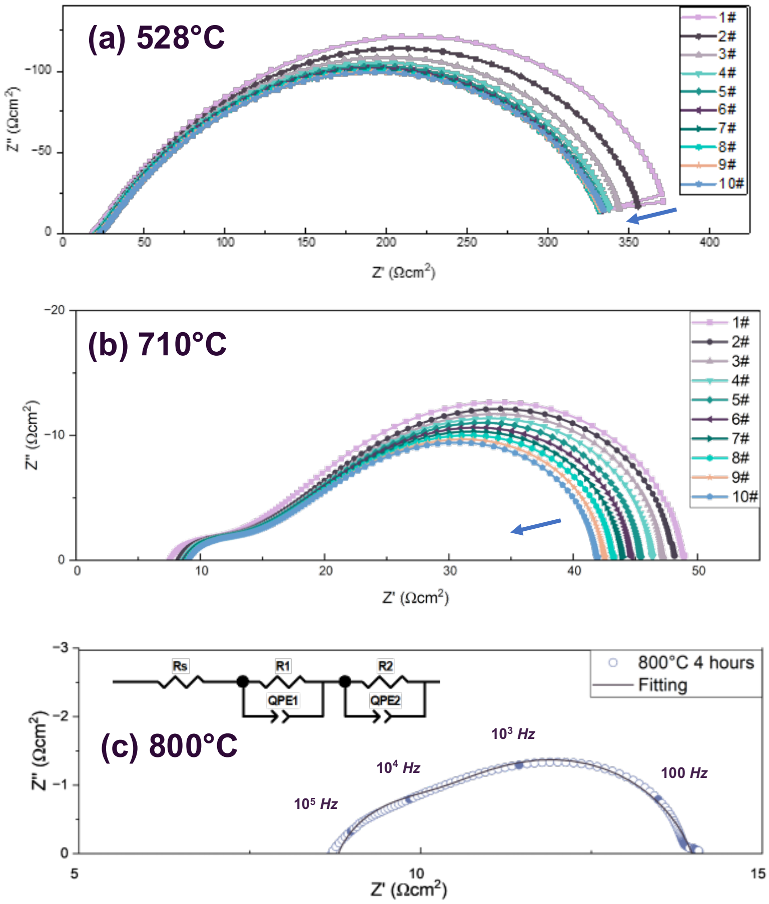

Figure S.10 EIS spectra of L1.85NTR/LSGM/L1.85NTR symmetric cell measured in flowing wet 5%H_2/Ar at 538°C(a), 710°C(b). The spectra were measured continuously with the reduction progressing. Each spectrum was scanned for 30mins. (c) EIS spectrum of symmetric cell measured after reduction in flowing wet 5%H_2/Ar for 4 hours at 800°C, and the fitted plots. The inserted sketches illustrate the equivalent circuit used in the fit.

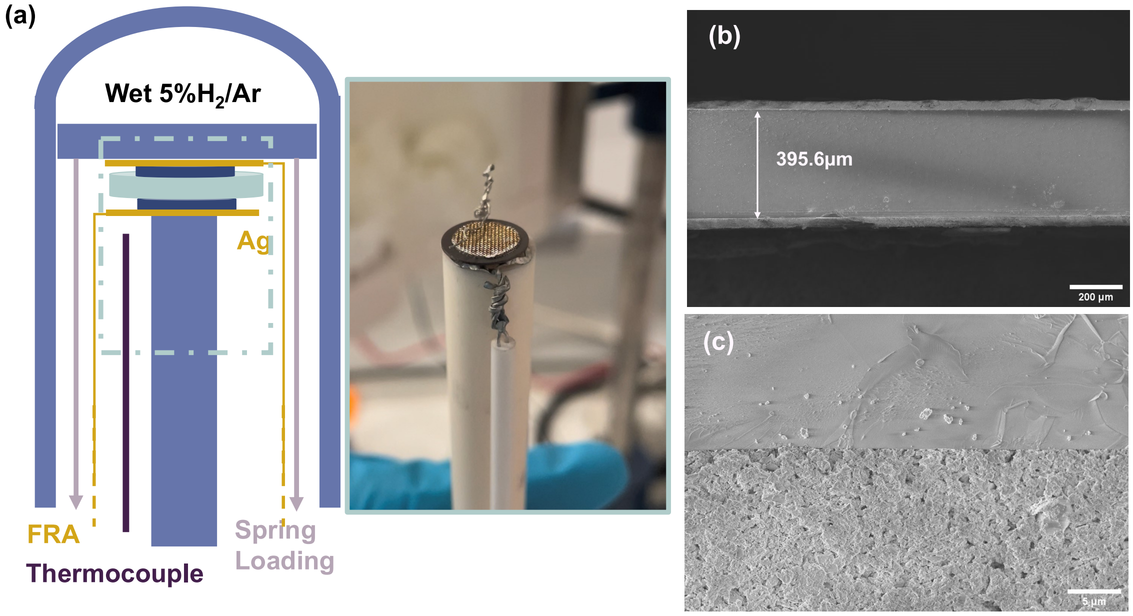

Figure S.11 (a) Schematic illustration of the testing rig for the EIS measurement; (b,c) SEM images of the microstructure of the L1.85NTR/LSGM/L1.85NTR symmetric cell.

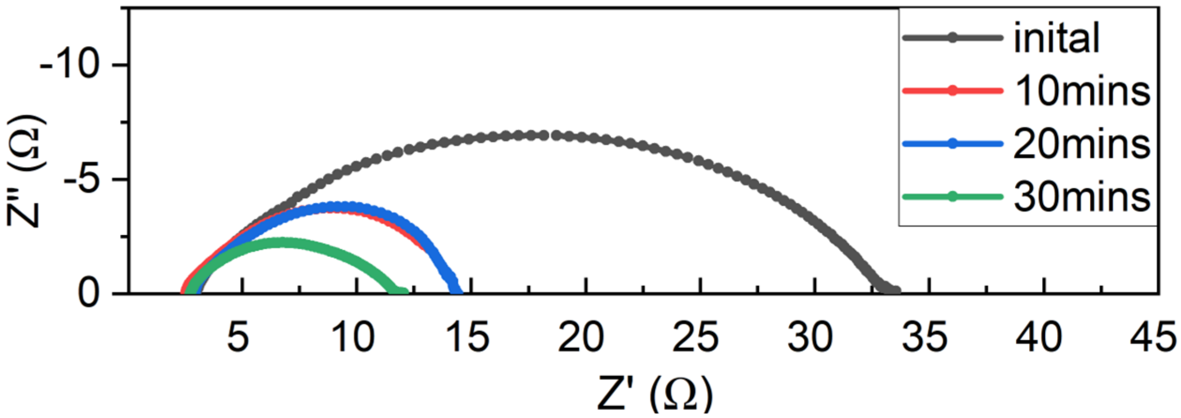

Figure S. 12 EIS spectra of the L1.85NTR/Hionic™ Electrolyte/LSM-GDC single cell during the reduction in wet H_2 (3% H_2O) at 850°C, confirming the exsolution of Ni nanoparticles of L1.85NTR electrode.

2. Tables

Table S.1 Goldschmidt tolerance factor and Le Bail refined lattice parameters of L2NT and L2NTR.

	Goldschmidt tolerance factor	a(Å)	b(Å)	c(Å)	β(°)
**L2NT**	0.9556	5.552(1)	5.559(1)	7.847(1)	90.05(1)
**L2NTR**	0.9552	5.552(2)	5.564(1)	7.852(1)	90.02(1)

Table S.2 ICP results of stoichiometric sample L2NT and L2NTR and A-site deficient sample L1.85NTR. B-site positions were assumed fully occupied by nickel, titanium, and ruthenium cations. Oxygen concentration is calculated based on experimental results of metal cations.

L_xNT(R)	Nominal composition	Concentration ratio			Experimental composition
		A/B cations	Ni/Ti-Ru	Ru/Ti	
L2NT	La_2NiTiO_6	1.04(2)	0.98(2)	0.0004(3)	$La_{2.08(5)}Ni_{0.98(2)}Ti_{1.01(8)}O_{6.12}$
L2NTR	$La_2NiTi_{0.9}Ru_{0.1}O_6$	1.03(1)	1.02(1)	0.11(1)	$La_{2.05(2)}Ni_{1.01(2)}Ti_{0.89(4)}Ru_{0.10(1)}O_{6.07}$
L1.85NTR	$La_{1.85}NiTi_{0.9}Ru_{0.1}O_6$	0.97(1)	0.99(1)	0.11(2)	$La_{1.93(1)}Ni_{0.99(1)}Ti_{0.90(1)}Ru_{0.10(1)}O_{5.885}$

Table S.3 Rietveld refined lattice parameters and atomic parameters of L1.85NTR

Lattice parameters and atomic parameters					
**a (Å)**			5.550(1)		
**b (Å)**			5.559(1)		
**c (Å)**			7.844(1)		
**β (°)**			90.046(1)		
	**x**	**y**	**z**	**B**	**Occupancy**
**La1**	0.5007(8)	0.5286(1)	0.249(7)	0.54(0)	1.888(2)
**Ti1**	0	0.5	0	0.680(3)	0.184(1)
**Ru1**	0	0.5	0	0.680(3)	0.020(1)
**Ni1**	0	0.5	0	0.680(3)	0.807(1)
**Ni2**	0.5	0	0	0.264(3)	0.192(1)
**Ti2**	0.5	0	0	0.264(3)	0.714(2)
**Ru2**	0.5	0	0	0.264(3)	0.080(1)
**O1**	0.214	0.198	-0.023	0.1	0.962
**O2**	0.279	0.727	-0.044	0.1	0.962
**O3**	0.423	0.989	0.274	0.1	0.962

Table S.4 Rietveld refined lattice parameter and atomic parameters of double perovskite phase in the reduced L1.85NR sample based on reference structure ICSD186433 [3].

Main phase lattice parameters and atomic parameters					
	**a (Å)**			5.550(1)	
	**b (Å)**			5.560(1)	
	**c (Å)**			7.845(1)	
	**β (°)**			90.055(1)	
	**x**	**y**	**z**	**B**	**Occupancy**
**La1**	0.50024(8)	0.52813(1)	0.25025(7)	0.508(1)	1.910(1)
**Ti1**	0	0.5	0	0.784(3)	0.226(1)
**Ru1**	0	0.5	0	0.784(3)	0.054(1)
**Ni1**	0	0.5	0	0.784(3)	0.770(1)
**Ni2**	0.5	0	0	0.175(5)	0.230(1)
**Ti2**	0.5	0	0	0.175(5)	0.674(1)
**Ru2**	0.5	0	0	0.175(5)	0.460(1)
**O1**	0.214	0.198	-0.023	0.1	0.962
**O2**	0.279	0.727	-0.044	0.1	0.962
**O3**	0.423	0.989	0.274	0.1	0.962

Three different reducing atmospheres were utilized in this work: wet 5%H_2/Ar, dry 5%H_2/Ar and high vacuum in microscope. The high vacuum condition in transmission electron microscope was considered

as 1.3×10^{-10} bar. The partial pressure of oxygen and hydrogen content were evaluated as 2.73×10^{-11} bar and 7.8×10^{-18} bar.

For the hydrogen gas, ESS quadrupole mass spectroscopy [4] was also used to confirm the difference between wet and dry 5%H_2/Ar:

Table S.5 mole fraction of oxygen, hydrogen and water calculated based the ionic current measured by quadrupole mass spectroscopy.

	**Wet 5%H_2/Ar**	**Dry 5%H_2/Ar**
Oxygen	0.0722mol%	0.0716mol%
Hydrogen	2.10 mol%	2.13mol%
Water	3.98 mol%	0.611 mol%

From Table S.5, the oxygen and hydrogen mole fraction of wet and dry 5%H_2/Ar are similar to each other. Hence, it's comparable to correlate the *in-situ* observation of mass loss in dry 5%H_2/Ar and the *ex-situ* observation after reduction in wet 5%H_2/Ar.

Table S. 6 Fitted parameters of the components in the equivalent circuits of the spectrum measured after reduction at 800°C in 5%H_2/Ar.

Temperature	Rs(Ωcm²)	R_1(Ωcm²)	QPE_1-Q	QPE_1-n	Capacitance(C_1,F)	R_2(Ωcm²)	QPE_2-Q	QPE_2-n	Capacitance(C_2,F)
800°C	8.79(2)	1.5(2)	$2.9(1)\times10^{-9}$	0.75(3)	7.8×10^{-6}	3.7(2)	$1.34(8)\times10^{-4}$	0.76(1)	1.93×10^{-5}

3. Equations

For double perovskite with two elemental cations occupying B-site positions, the ordering of B-cations was assessed through the degree of ordering, S[5]:

$$S = 2g_B - 1 = 0.616 \qquad S1$$

where g_B represents the correct occupancy of Ni or Ti-Ru cations at either the (0, ½, 0) or (½, 0, 0) positions in this work.

For thermogravimetric analysis, Amaya-Dueñas et al.[6] characterized the Ni exsolution from $La(Sr)Cr_{0.85}Ni_{0.15}O_{3-\delta}$ with TGA in 5%H_2/Ar and calculated the oxygen deficiency, δ. The exsolution and decomposition processes can be assumed as a subsequent reaction after oxygen loss. Consequently, according to the method described, the loss of oxygen can be derived using Equation S2 below:

$$l_O = \frac{MW_{L1.85NTR}}{A_O} \Delta m \qquad S2$$

where $M_{L1.85NTR}$ is the molecular weight of L1.85NTR. Δm is the measured mass loss from TG curve. A_O is the atomic mass of oxygen and l_O is the loss of oxygen. Based on the Rietveld refinement result, if we assume the molecular formula of L1.85NTR as $La_{1.888}NiTi_{0.9}Ru_{0.1}O_{5.832}$, the l_O of the sample reduced at 800°C is approximately 0.25 mol per mol of L1.85NTR.

Reference

1 Hk. Müller-Buschbaum and H. G. V. Schnering, *ZAAC - Journal of Inorganic and General Chemistry*, 1965, **340**, 232–245.

2 H. E. Swanson and E. Tatge, *Standard X-ray Diffraction Powder Patterns*, 1966.

3 W. Z. Yang, X. Q. Liu, Y. Q. Lin and X. M. Chen, *J Appl Phys*, , DOI:10.1063/1.4704392.

4 ESS Ltd, CatalySys, https://www.essco.com/catalysys.

5 S. Vasala and M. Karppinen, *Progress in Solid State Chemistry*, 2015, **43**, 1–36.

6 D. M. Amaya-Dueñas, G. Chen, A. Weidenkaff, N. Sata, F. Han, I. Biswas, R. Costa and K. A. Friedrich, *J Mater Chem A Mater*, 2021, **9**, 5685–5701.
